# Voice Your Values, Tailored Advance Care Planning in Persons Living With Mild Dementia: A Feasibility Study

**DOI:** 10.1093/geroni/igab046.1561

**Published:** 2021-12-17

**Authors:** Shirin Vellani, Martine Puts, Katherine McGilton, Andrea Iaboni

**Affiliations:** 1 McMaster University, Toronto, Ontario, Canada; 2 University of Toronto, University of Toronto/Toronto, Ontario, Canada; 3 KITE-Toronto Rehabilitation, University Health Network, Toronto, Ontario, Canada; 4 Toronto Rehabilitation Institute, University Health Network, Toronto, Ontario, Canada

## Abstract

Older adults diagnosed with mild dementia can identify their wishes, values and goals of care with a high degree of accuracy and reliability. However, there is a paucity of research to guide best practices on how to incorporate Advance Care Planning (ACP) in the care of older adults living with mild dementia. Thus, only a minority of them participate in any ACP discussions. We developed an intervention called Voice Your Values (VYV) that healthcare professionals can implement to identify and document values of older adults. This single group pretest and posttest design aimed to determine the feasibility, acceptability and preliminary efficacy of the intervention. A convenience sample of 20 dyads of older adults and their trusted individuals were recruited from 4 geriatric clinics. Tailored VYV intervention was delivered to dyads on a one-on-one basis over two sessions using videoconferencing. Feasibility was determined through recruitment and retention rates, and intervention fidelity. Acceptability was assessed using modified Treatment Evaluation Inventory. Primary outcome was the Surrogate Decision-Making Confidence Scale. Secondary outcomes included an ACP engagement survey to assess older adults’ engagement in ACP; Dementia Knowledge Assessment Tool for trusted individuals; and the Kessler Psychological Distress Scale for all participants. The recruitment rate was 45%, retention rate was 100% and 92% participants rated VYV as highly acceptable. Trusted individuals showed statistically significant improvement in decision-making confidence (p=.02) and psychological distress (p=.02); but no improvement in dementia knowledge (p=.47). Older adults demonstrated statistically significant improvement in ACP engagement (p=<.01). Initial feasibility of VYV was demonstrated.

